# ResNet-Locust-BN Network-Based Automatic Identification of East Asian Migratory Locust Species and Instars from RGB Images

**DOI:** 10.3390/insects11080458

**Published:** 2020-07-22

**Authors:** Sijing Ye, Shuhan Lu, Xuesong Bai, Jinfeng Gu

**Affiliations:** 1 State Key Laboratory of Earth Surface Processes and Resource Ecology, Beijing Normal University, Beijing 100875, China; 2 Center for Geodata and Analysis, Beijing Normal University, Beijing 100875, China; 3 Master of Health Informatics, School of Information, University of Michigan, Ann Arbor, MI 48109, USA; 4 Key Laboratory of Agricultural Land Quality (Beijing) Ministry of Land and Resources, China Agricultural University, Beijing 100083, China; xs.bai@cau.edu.cn (X.B.); jf.gu@foxmail.com (J.G.)

**Keywords:** locust, grasshopper, monitoring and forecasting, image processing, deep learning, CNN

## Abstract

Locusts are agricultural pests found in many parts of the world. Developing efficient and accurate locust information acquisition techniques helps in understanding the relation between locust distribution density and structural changes in locust communities. It also helps in understanding the hydrothermal and vegetation growth conditions that affect locusts in their habitats in various parts of the world as well as in providing rapid and accurate warnings on locust plague outbreak. This study is a preliminary attempt to explore whether the batch normalization-based convolutional neural network (CNN) model can be applied used to perform automatic classification of East Asian migratory locust (AM locust), *Oxya chinensis* (rice locusts), and cotton locusts. In this paper, we present a way of applying the CNN technique to identify species and instars of locusts using the proposed ResNet-Locust-BN model. This model is based on the ResNet architecture and involves introduction of a BatchNorm function before each convolution layer to improve the network’s stability, convergence speed, and classification accuracy. Subsequently, locust image data collected in the field were used as input to train the model. By performing comparison experiments of the activation function, initial learning rate, and batch size, we selected ReLU as the preferred activation function. The initial learning rate and batch size were set to 0.1 and 32, respectively. Experiments performed to evaluate the accuracy of the proposed ResNet-Locust-BN model show that the model can effectively distinguish AM locust from rice locusts (93.60% accuracy) and cotton locusts (97.80% accuracy). The model also performed well in identifying the growth status information of AM locusts (third-instar (77.20% accuracy), fifth-instar (88.40% accuracy), and adult (93.80% accuracy)) with an overall accuracy of 90.16%. This is higher than the accuracy scores obtained by using other typical models: AlexNet (73.68%), GoogLeNet (69.12%), ResNet 18 (67.60%), ResNet 50 (80.84%), and VggNet (81.70%). Further, the model has good robustness and fast convergence rate.

## 1. Introduction

Locusts are agricultural pests found in many parts of the worldwide [1]. Globally, approximately 50 million km^2^ of the total land area is infested by locusts each year, and about one-eighth (1/8) of the world’s population is affected by locust plagues [2]. In the last 2500 years, China has suffered from frequent locust plagues; the extent and severity of which have been the highest in the world. Of all locust types, East Asian migratory locusts (AM locusts), which appear suddenly and are migratory in nature, cause the most severe damage [3]. For example, in September 2017, AM locusts wiped out nearly 66 ha of crops in Shandong Province, and in July 2015, an AM locust plague covering an area of nearly 8200 km^2^ occurred in the city of Chifeng in the Inner Mongolia autonomous region. Therefore, rapidly and accurately acquiring information concerning the growth conditions, distribution densities, and community features of locusts are important for the timely and effective prevention and control of locust plagues.

Conventional locust monitoring systems rely on manual field acquisition of the locust type, instar, and population data obtained from plant protection specialists. This method has numerous shortcomings, including the high cost of data acquisition, low efficiency, limited coverage, inability to monitor some locust-infested areas in the field (e.g., lakes, wetlands, and tidal flats), difficulties in identification (locust instars are generally identified based on the development of their wings; however, third-, fourth-, and fifth-instar locusts do not have notably different wings and thus cannot be effectively distinguished by specialists), and the proneness to identification errors owing to the existence of numerous locust types and morphologies as well as their complex habitats [4]. Additionally, because the locusts can increase rapidly in number and migratory, manual surveys are unable to respond to rapid changes in the locust distribution density and community features. Given these challenges, several researchers have increased their monitoring scales [5,6]. Specifically, they have shifted from monitoring individual locusts and locust communities on a quadrat scale to monitoring host vegetation and habitats of locusts on a regional scale. These researchers have banked on the advantages of satellite remote-sensing observation technologies (e.g., wide-coverage, high temporal, and spatial resolutions) to make up for the deficiencies of conventional methods [7,8,9,10]; this has resulted in excellent results reported by several researchers [11]. As early as 1986, Bryceson and Wright [12] proposed combining a locust breeding simulation model and satellite remote-sensing data to extract areas suitable for locust breeding by monitoring changes in the vegetation; they also demonstrated that using this approach could effectively supplement missing ground survey data. Bryceson et al. [13] applied digital satellite data to detect potential areas of Australian plague locust outbreaks at an early stage in semi-arid regions of Southwest Queensland. Waldner et al. [14] produced dynamic greenness maps by developing a colorimetric transformation from SPOT-VEGETATION and moderate resolution imaging spectroradiometer (MODIS) data and used them in day to day monitoring of desert locusts in arid—semi-arid regions. This method was found to be exhibit significant accuracy when employed in summer breeding areas; however, the accuracy decreased when employed in winter breeding areas.

Further, researchers noted that the current spatial resolution remains too coarse to resolve complex or fragmented patterns [14]. Shi et al. [15] extracted seasonal characteristics of typical land covers in normalized difference vegetation index, triangular vegetation index, and land surface temperature data from fused Landsat-MODIS surface reflectance imagery data. Based on the results they obtained, they proposed a landscape membership-based random forest algorithm to quantify landscape structure and hydrological regimen of locust habitats at the patch level. Gómez et al. [16] extracted surface temperature, leaf area index, and soil moisture root zones from the soil moisture active passive satellite data as environmental factors and analyzed their correlations with species’ occurrences of desert locusts using species distribution models. However, developing satellite remote-sensing observation techniques alone cannot meet the requirements for rapid and accurate locust plague prevention and control. Satellite remote-sensing observations are affected by factors such as orbits and weather conditions and, therefore, cannot meet the long-duration and high-timeliness requirements of locust monitoring systems. Also, the key to using satellite remote-sensing technologies to monitor and provide warnings of locust plagues is by recognizing the relation between locust distribution density and structural changes in locust communities using the hydrothermal and vegetation growth conditions in their habitats at various regions. This requires large amounts of quadrat-scale locust instar, type, and distribution density observation data. Additionally, sample scale locust observation data are necessary in understanding the occurrence processes of locust plagues and their driving factors. Therefore, developing efficient and accurate sample scale locust information acquisition techniques plays a significant role in understanding the occurrence processes [17] and causes of locust plagues and providing rapid and accurate warnings about locust plagues.

Recently, computer-vision techniques have developed rapidly and have been extensively applied in monitoring and identification of insects [18,19]. Goto et al. [20] proposed a rapid computer-vision method for monitoring adult beetles and evaluating their numbers. Drake et al. [21] successfully extracted segmented images of target individuals and rapidly identified and classified a large number of specimens by equipping an insect identification system developed for the United States Department of Agriculture with an image segmentation algorithm. By improving the image identification rate by employing scale-invariant feature transforms and the LOSS algorithm, Solis-Sánchez et al. [22] identified various pests, including *Diabrotica* (Coleoptera: Chrysomelidae), *Aphis gossypii* Genn., *Thrips tabaci* L. (adult), and *Bemisia tabaci* Genn., with high accuracy. Cai et al. [23] proposed a reverse identification method for identifying pests based on leaves eaten by pests, extracted eigenvalues, and constructed a backpropagation (BP) neural network identification model. Additionally, they demonstrated that the proposed method outperforms previous methods in terms of identification efficiency and accuracy. Zhang [24] extracted morphological and color features of eight common farmland pests used a backpropagation (BP) neural network classifier to classify the pests and achieved an accuracy of 85.70%. Zheng et al. [25] identified locusts and calculated population densities by dynamically extracting color, area, and morphological features by employing an image processing technique. Xiong et al. [26] proposed a locust identification model based on near-infrared spectroscopy and hierarchical clustering. They examined the feasibility of this model for rapid detection of locusts in complex environments with interlaced vegetation, soil, and rocks and found that their model achieved an accuracy of up to 91.67%. Zhang et al. [27] formulated an improved split-merge algorithm by introducing a pulse-coupled neural network and a simplified Mumford-Shah model and applied it to the segment images of adult locusts and locust larvae. Zhang [28] proposed a method that involves combining a maximum-similarity region-merging algorithm with texture and color histograms to identify pests. The results the obtained shows that the algorithm they proposed was able to extract target pests against a complex farmland background satisfactorily. Chen et al. [29] constructed a pest identification system by integrating feature learning, feature fusion, classification, and position regression of 16 kinds of common pests based on deep learning combineds. The recognition accuracy of the proposed method in light traps, under natural conditions, ranged from 66.00 to 90.00%. Thenmozhi et al. [30] proposed an efficient deep CNN model to classify insect species on three publicly available insect datasets [31,32] and achieved an accuracy of 96.75, 97.47, and 95.97% respectively. However, most of these studies primarily focused on obtaining the quantities of various types of insects. Few studies have been conducted to achieve automatic acquisition of locust type and instar information in field environments.

In this paper, we apply CNN for the identification of species and instars information of locusts using our proposed ResNet-Locust-BN model. Based on the ResNet architecture, we integrate the BatchNorm function before each convolution layer to improve network stability, convergence speed, and classification accuracy. Subsequently, locust image data collected in the field were used as sample inputs for training the classifier parameters. We performed comparison experiments to optimize the activation function, initial learning rate, and batch size model parameters of the proposed model. We identified ReLU as the most appropriate activation function for the layers of the proposed model. The initial learning rate and batch size were set to 0.1 and 32, respectively. Results of various experiments performed show that the ResNet-Locust-BN network model can distinguish AM locusts from *Oxya chinensis* (rice locusts) and cotton locusts with high accuracy. We also observed that the proposed model can identify the growth status information of AM locusts (third-instar, fifth-instar, and adult) with an overall accuracy of 90.16%, which is higher than the accuracies reported for the AlexNet, GoogLeNet, ResNet 18, ResNet 50, and VggNet CNN models. Further, the proposed model showed good robustness, fast convergence, and other advantages.

The remaining part of this paper is organized as follows: Section 2 provides the details of the experimental datasets, differences in locust phenotypic characteristics, the architecture of the proposed ResNet-Locust-BN network model, and the experimental process. Section 3 discusses the optimization of the experimental parameters and recognition accuracy of different CNN models. Section 4 provides discussions on comparison of relevant studies, shortcomings, and our intended future work with respect to this study. Finally, Section 5 presents the conclusions of this study.

## 2. Data and Methods

### 2.1. Experimental Datasets

**Figure 1:**
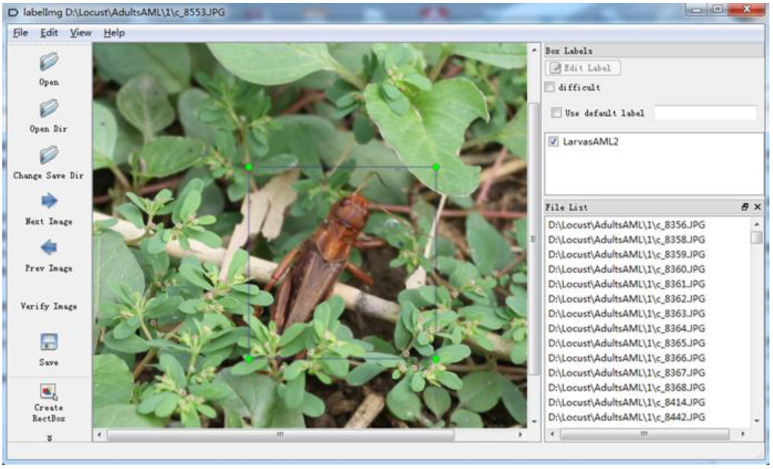
Schematic diagram of sample annotation based on lableImg.

CNNs extract a large number of abstract features of target elements via multi-layer convolution calculations to achieve high accuracy in image classification tasks. Therefore, a large number of parameters need to be solved, which require the input of a large number of samples in the training process to improve the reliability and robustness of the classification model. We constructed outdoor test areas (including grass, soil and crops, rocks, and other objects), put in third-instar, fifth-instar, and adult AM locusts, adult rice locusts, and adult cotton locusts (close to 2000 for each type) to obtain a sufficient number of training samples. Thus, we collected images of locusts with different poses (because specimen appearance varies with the orientation they are being shown) and background using a visible light camera under the condition of “normal lighting and shooting.” Then, an open-source software “labelImg” was applied to manually label the locust location border information and species’ information on the original images to generate XML files, as shown in Figure 1. In this regard, each of the original images was cut into several sample blocks according to the content of a corresponding XML file (each sample block corresponds to one locust); and all sample blocks were extracted and stored in a corresponding sample dataset. Particularly, we manually obtained 6000 sample blocks for each type of locust, i.e., a total of 30,000 sample blocks were extracted, which took one of our co-authors around 300 h. Afterward, we divided the dataset (in a random manner) into training, validation, and test datasets. To be specific, the training set contained 4000 samples, the validation set contained 1000 while the test set contained 1000 samples as shown in Table 1.

### 2.2. Differences in Locust Phenotypic Characteristics

**Figure 2:**
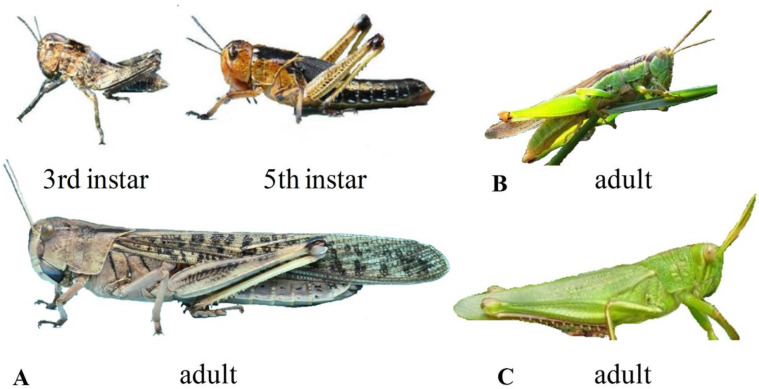
Differences of external morphology among different species and instars. (**A**) presents AM locusts; (**B**) presents rice locusts; and (**C**) presents cotton locusts.

Locusts belong to the order Orthoptera, which has nymph and adult growth stages, where the nymph stage can be divided into several sub-stages. The purpose of our research is to realize the automatic identification of AM locusts (third-instar, fifth-instar, and adult stage), rice locusts (adult stage), and cotton locusts (adult stage). The body compositions of the research objects are consistent; however, there are significant differences in their external morphology [33], as Figure 2 shows.

As shown in Table 2, the body size and wing length of adult cotton locusts are significantly larger than those of the other two species. Their body color is yellow-green, their front and back plates are angular and slightly protruding, while their antennae are filamentous. Second, compared to AM locusts, rice locusts have a smoother exterior and backplate, a squarer head, a more slender antennae, a pointed web, and a green or brown-green body-color (similar to the color of rice). Third, there are significant differences in the body length, wing tooth length, and antennae segment number for different instars of AM locusts. For the third-instar, there are approximately 20–21 antennae segments, the body length is 10–20 mm, the wing tooth length only reaches the first abdominal section, and the posterior wing teeth are slightly triangular. For the fifth-instar, there are approximately 24–25 antennae segments, the body length is 26–40 mm, the wing teeth are obviously developed, and the length covers the fourth to fifth abdominal segments. The adult body lengths show significant male and female differences, with a male body length of 35–42 mm and a female body length of 39–52 mm.

In summary, the research objects in this paper have differences in body shape, shape, and color of wing teeth, and other aspects, which meet the requirements for automatic classification. Note that swarming (gregarious) AM locusts and scattered (dispersed) AM locusts often show different color characteristics. The sampled image data in this paper are all swarming AM locusts.

### 2.3. ResNet-Locust-BN Network

In recent years, CNN models have been widely used in the field of image processing. They have achieved excellent results in image classification, target detection, semantic segmentation, and video processing [34,35,36]. In general, the CNN network structure extracts abstract image features via convolution and pooling processes at many levels, integration, and reduction of dimensions with full connection processes, and provides a nonlinear modeling capability with different activation functions. On this basis, a large number of sample images are used as input, and, via BP, the gradient descent algorithm can be performed to iteratively modify the model parameters. Finally, it constructs a classifier oriented to a specific target. However, can CNN models accurately identify AM locust species and instars in the wild? To solve this problem, we selected five typical CNN models (the network structure is the same as the one used by the original author of the model) at the beginning of this study. Each model showed excellent accuracy even when designed with different characteristics. AlexNet proposed data augmentation, dropout method, and provided a pioneering contribution to the development of deep learning. Similarly, VggNet uses a convolutional layer combining small filters to express more powerful features of the input data with fewer parameters. In GoogLeNet, inception-based modular architecture was employed instead of increasing network depth to enhance the adaptability of the network to multiple scales and improve training accuracy. ResNet provided an available solution to vanishing gradient problem that results from network depth increase. In this study, we used both ResNet18 and ResNet50 as experimental models because ResNet18 and ResNet50 have different layer structures. ResNet18 is only layered with basic building block, and ResNet50 has a structure of BottleNeck building block with an additional layer of 1 × 1 convolution, which could reduce and restore the dimensions. He [37] pointed out that increasing the layers of training network only will not decrease training error correspondingly. He also noted that doing so could result in a degradation problem. To be more precise, it is necessary to list both the testing result for ResNet18 and ResNet 50 as well. The above-mentioned sample dataset was used as the model input, and the classification tests of the locust species and instars were directly performed. The experimental results obtained are shown in Table 3, where the experimental accuracy is maximized by modifying the batch size, activation function, initial learning rate parameters, and conducting multiple experiments. The results from the experiments performed show that CNN models have excellent applicability in species and instars recognition of locusts from visible field images. Out of all the models tested, ResNet50 produced the highest accuracy rate of approximately 80.84%. For each CNN model, the accuracy was calculated as the ratio of the number of correctly identified test samples to the total number of test samples.

However, can we further improve the classification accuracy by optimizing the network structure? First, we maintain that owing to the influence of environmental changes in the field image collection and individual differences between different instars and different species of locusts, large differences between different sample data is an important factor affecting the classification accuracy. Therefore, the classification accuracy can be effectively improved by dynamically improving the consistency of the data. Second, as the number of network layers deepens, a gradient discretization or gradient explosion may be triggered which could reduce the robustness and stability of the locust image feature extraction. Third, because the distribution of each layer’s inputs changes as the parameters of the previous layers change, the training process requires a lower learning rate and careful parameter initialization, which makes it notoriously hard to train models with saturating nonlinearities. Common methods to solve this problem include increasing the number of training samples to match more data with the model parameters or discarding some random or specific features in the training process to reduce the number of model parameters. However, it is worthy of note that these methods often have difficulties in improving classification accuracy. Batch normalization [38] provides a good solution to this problem, which tolerates higher learning rates and less carefulness in parameter initialization. 

According to Ioffe’s test, batch normalization achieves the same accuracy with 14 times fewer training steps [38]. Shang et al. [39] verify that the batch normalization method helps to distribute representation learning to residual blocks at all layers, as opposed to a plain ResNet that does not involve batch normalization, in which case learning happens mostly in the latter part of the network. The authors also showed that batch normalization can effectively regularize a concatenated ReLU activation scheme on ResNets, whose magnitude of activation grows by preserving both positive and negative responses when going deeper into the network. 

**Figure 3:**
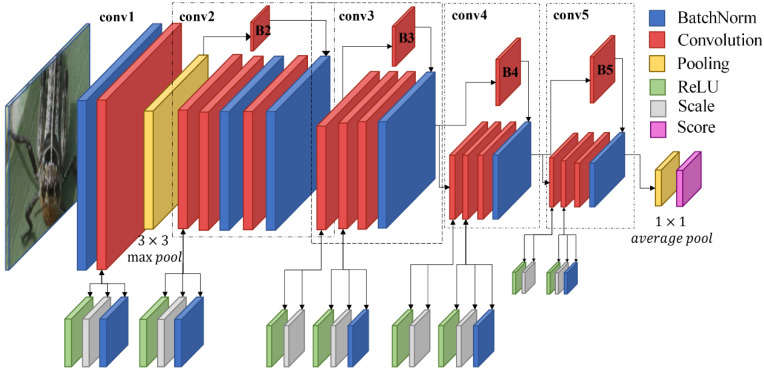
The logical structure of the ResNet-Locust-BN network. Note: Conv1-5 presents five groups of primary convolution process with different parameters and number of iterations. Convolution parameters in sequence of: conv1 is [7 × 7 (kernel size), 64 (channels number), *s* (stride) = 2, *p* (pad) = 3]; conv2 are {[1 × 1, 64, *s* = 2, *p* = 0], [3 × 3, 64, *s* = 2, *p* = 1], [1 × 1, 256, *s* = 2, *p* = 0]} × 3 (number of iterations); conv3 are {[1 × 1, 128, *s* = 2, *p* = 0], [3 × 3, 128, *s* = 2, *p* = 1], [1 × 1, 512, *s* = 2, *p* = 0]} × 4; conv4 are {[1 × 1, 256, *s* = 2, *p* = 0], [3 × 3, 256, *s* = 2, *p* = 1], [1 × 1, 1024, *s* = 2, *p* = 0]} × 6; conv5 are {[1 × 1, 512, *s* = 2, *p* = 0], [3 × 3, 512, *s* = 2, *p* = 1], [1 × 1, 2048, *s* = 2, *p* = 0]} × 3. Parameters of shortcut convolution B2, B3, B4, B5 are respectively [1 × 1, 256, *s* = 2, *p* = 0], [1 × 1, 512, *s* = 2, *p* = 0], [1 × 1, 1024, *s* = 2, *p* = 0], [1 × 1, 2048, *s* = 2, *p* = 0].

These studies are of great significance and provided deep inspiration for this study [38,39,40,41]. In this paper, we propose a ResNet-Locust-BN model base on ResNet50 architecture [37]. The batch normalization function [38] is integrated before each convolution layer of the proposed model to perform normalization calculations. This makes it possible to increase the relativity of dataset, reduce the absoluteness among sample blocks, and improve the classification accuracy. Further, it can also ensure a more stable operation of the network and improve the robustness of the model. Further, by introducing batch normalization function, ResNet-Locust-BN can set a sizeable initial learning rate without worrying about gradient dispersion and, thus, achieve a faster model convergence. The structure of the ResNet-Locust-BN model is shown in Figure 3.

### 2.4. Experimental Process

**Figure 4:**
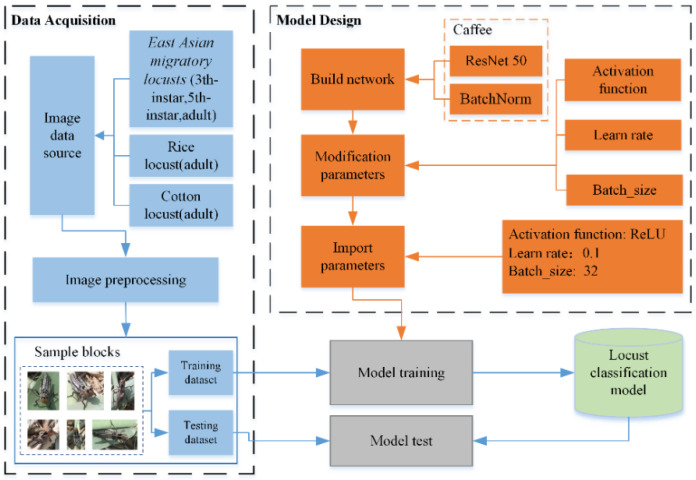
Experimental process of locust image classification.

The experimental process involved three stages, namely, data acquisition, model design, and model testing, as shown in Figure 4. In the data acquisition stage, we collected images of locusts of various types and instars outdoors, performed image processing (including locust frame, type and instar information labeling, cropping, image graying, and histogram equalization enhancement), and constructed a locust classification sample dataset (total number of samples: 30,000). The model design stage involved designing the network structure, selecting the model parameters, and developing the model. In the model test stage, we entered the training dataset, implemented the model training process, constructed the locust classification model, and applied the testing dataset to test the accuracy of the model. Table 4 lists the details of the environment of the experimental infrastructure.

## 3. Results

### 3.1. Optimization of the Experimental Parameters

**Figure 5:**
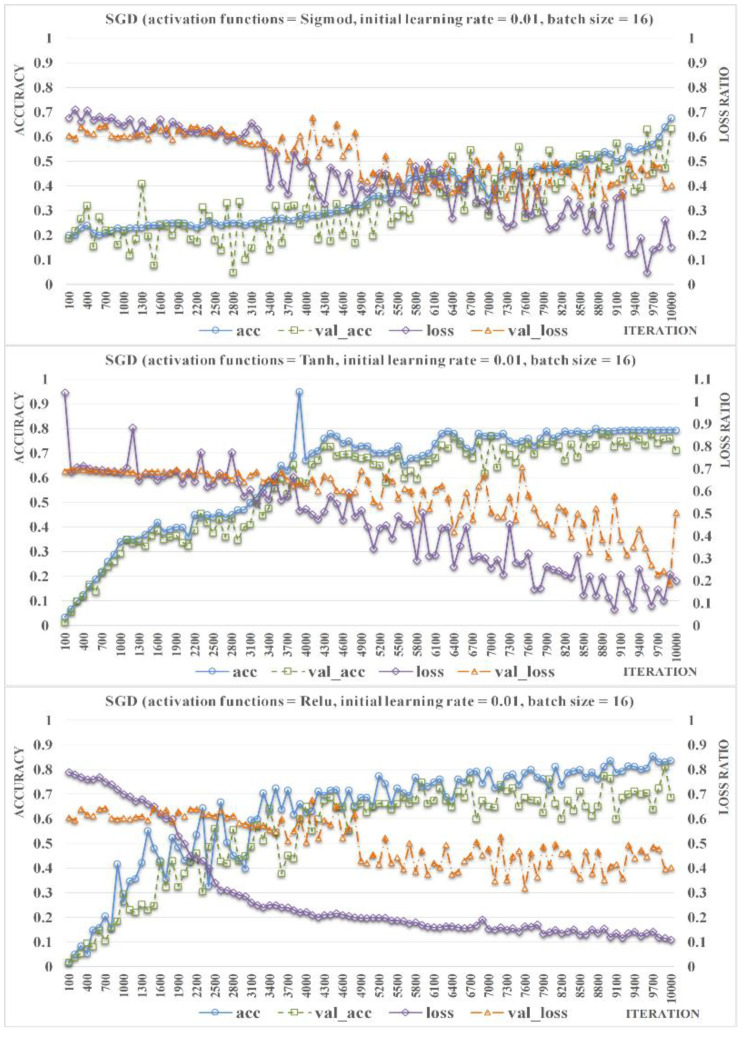
Comparison of the accuracy rate and the loss rate of ResNet-Locust-BN based on different activation functions (initial learning rate = 0.01, batch size = 16).

In this paper, different activation functions, initial learning rates, and batch sizes were tested for 10,000 iterations via comparative experiments to evaluate their influence on the accuracy of the proposed ResNet-Locust-BN model. This is to get insight on how to optimize the settings of the model parameters. Stochastic gradient descent optimizer was used with default parameters (momentum = 0, decay = 0.0) in the experiments conducted. First, model accuracy and loss ration under the Sigmod, Tanh, and ReLU activation functions were calculated and listed based on the training and validation datasets as shown in Figure 5. Initial learning rate and batch size were set as 0.01 and 16, respectively. The accuracy (acc) is calculated as the ratio of the number of correctly identified samples to the total number of samples in the training dataset. The validation_accuracy (val acc) is calculated as the ratio of the total number of correctly identified samples to the total of samples in the validation dataset. The loss and val_loss refer to loss ratio of training and validation datasets, respectively. As the experimental result shows, the suitability of the Sigmod activation function is significantly lower than other activation functions as it resulted in lower acc, val_acc, and slower convergence rate of val_loss. ReLU activation function produced higher and more stable acc than Tanh. The val_acc of ReLU is similar to that of Tanh, but it exhibited better stability. Although loss obtained in the case of ReLU shows a faster convergence rate than that of Tanh, the change characteristics of its val_loss is similar to that of Tanh function. Hence, we selected ReLU as the model activation function as it exhibited better accuracy and stability.

**Figure 6:**
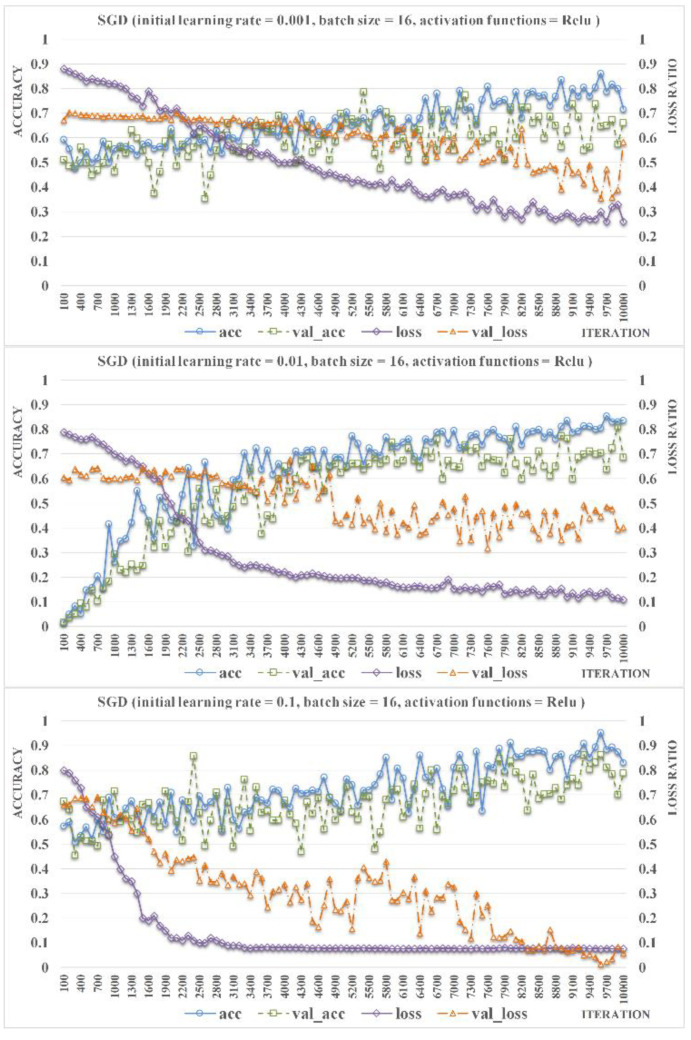
Comparison of the accuracy rate and the loss rate of ResNet-Locust-BN based on different initial learning rates (activation function = ReLU, batch size = 16).

Further, the advantages of introducing the batch normalization method [38] are to make the model compatible with larger initial vectors and to speed up the model convergence theoretically. In this study, we compared three different initial vector loss values, 0.001, 0.01, and 0.1, to improve the model accuracy, as shown in Figure 6. Batch size and activation function were set as 16 and ReLU, respectively. The experimental results show that increasing the initial learning rate from 0.01 to 0.1 significantly accelerated the convergence rate of both loss and val_loss. The small change of initial learning rate (i.e., from 0.001 to 0.01) has slight influence on the acc and val_acc. It is worthy of note that the acc and val_acc significantly improved as the initial learning rate was adjusted from 0.01 to 0.1. Furthermore, no overfitting phenomenon occurred during 10,000 iterations in these experiments by applying the batch normalization method. Hence, it is appropriate to set an initial learning rate as 0.1, by which both the accuracy rate and the loss rate can be improved.

**Figure 7:**
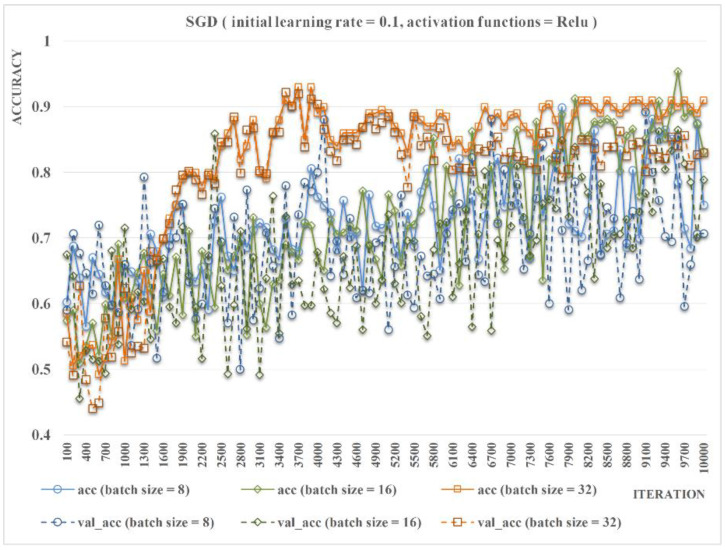
Comparison of the accuracy of ResNet-Locust-BN based on different batch sizes (activation function = ReLU, initial learning rate = 0.1).

Finally, we set the batch size to 8, 16, and 32 and calculated the changes in the recognition accuracy and the loss rate under corresponding conditions. As shown in Figure 7, change of batch size has little influence on the final identification accuracy of the model after thousands of iterations. However, as the batch size increases from 8 to 32, the fluctuation intensity decreased and the convergence rate of accuracy (acc and val_acc) increases. According to the experimental results we obtained, when the batch size is 32, the recognition accuracy is the highest and most stable with the fastest convergence rate. Because excessive batch size may reduce the generalization ability of the model and limited by the hardware performance, the optimal batch size was set as 32. Therefore, the activation function, initial learning rate, and batch size of ResNet-Locust-BN were set as ReLU, 0.1, and 32, respectively.

### 3.2. Recognition Accuracy of Different CNN Models

**Figure 8:**
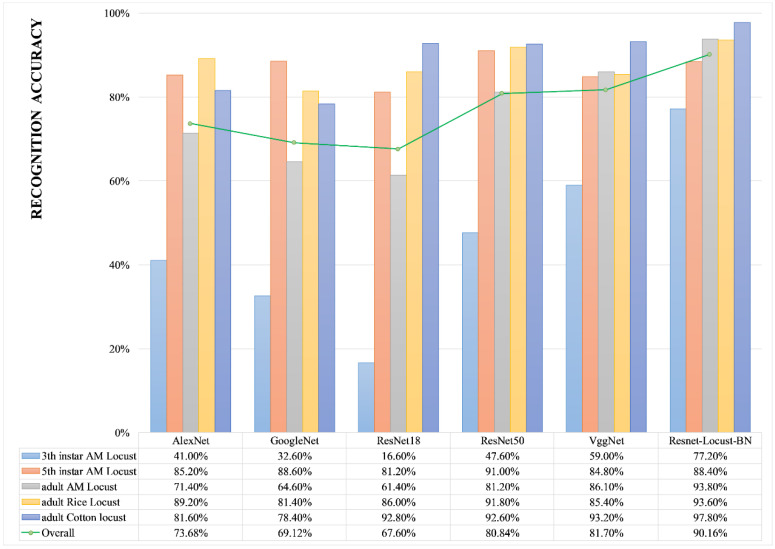
Accuracy comparison of the six CNN locust identification models.

In this study, we used the training dataset as input to build a classifier model based on six CNN models, which are as follows: AlexNet, GoogLeNet, ResNet 18, ResNet 50, VggNet, and ResNet-Locust-BN. Afterward, we used the test dataset to evaluate the accuracy of each model for the identification of AM locusts (third-instar, fifth-instar, and adult), rice locusts (adult), and cotton locusts (adult), as shown in Figure 8. For each CNN model, the accuracy was calculated as the ratio of the number of correctly identified test samples to the total number of test samples. The experimental results show that the six models evaluated generally exhibited high accuracies in the identification of rice locusts and cotton locusts. In contrast, identification of AM locusts (especially the third-instar AM locust) was more complicated. AlexNet, GoogLeNet, and ResNet 18 have a low recognition accuracy for third-instar and adult AM locusts and do not meet the application requirements. ResNet50, VggNet, and ResNet-Locust-BN present higher accuracy for the identification of fifth-instar AM locusts, adult East AM locusts, adult rice locusts, and adult cotton locusts. The ResNet50 model when tested with fifth-instar AM locust, produced a recognition accuracy rate of 91.00%, which slightly higher than the accuracy of ResNet-Locust-BN (88.40%) proposed in this paper. However, because ResNet-Locust-BN has a significantly higher recognition accuracy for third-instar AM locusts (77.20%) than other models, and has the highest overall accuracy (90.16%), we believe that ResNet-Locust-BN has better stability and applicability than the other models.

**Figure 9:**
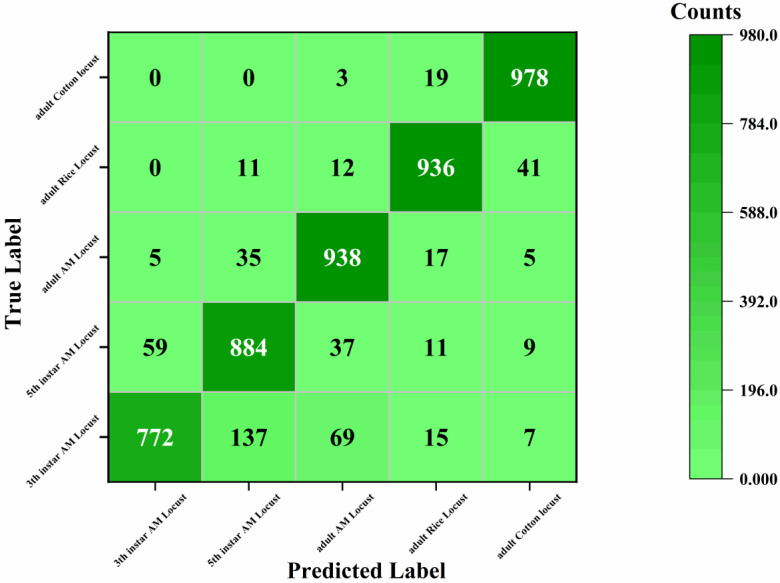
Confusion matrix base on ResNet-Locust-BN model.

We constructed a confusion matrix to present a more detailed description accuracy of the proposed ResNet-Locust-BN model in recognizing AM locusts, rice locusts and cotton locusts base on as shown in Figure 9. The recognition accuracy of adult locusts is generally higher than 93.50% and thus, can be considered adequate with respect to the application requirements. The true positive rate of the model in identifying these pests is 93.80%, 93.60%, and 97.80%, respectively. The low recognition accuracy of third-instar AM locusts is the greatest shortcoming of the proposed ResNet-Locust-BN model as well as other CNN models. Almost all erroneous judgments of third-instar and fifth-instar AM locusts are made in identifying AM locust with different instars. The third-instar AM locusts are more likely to be misidentified as fifth-instar AM locusts (with a corresponding error recognition rate of 10.70%), and vice versa (with a corresponding error recognition rate of 5.90%).

It seems that a key factor limiting the recognition accuracy of third-instar AM locusts is insufficient information of the RGB images generated in visible wavelengths rather than the CNN models. Hence, to improve the recognition rate, multispectral or hyperspectral technology could be integrated into the model to explore more significant differences among AM locust with different instars. For AM locusts with each instar type (third-instar, fifth-instar, and adult), several samples were misidentified as rice locusts (adult) or cotton locusts (adult), which may be due to interference by grass, soil, crops, rocks, and other objects in the outdoor test areas. Few rice locusts (adult) and cotton locusts (adult) samples were misidentified as AM locusts. Further, rice locusts (adult) are more likely to be misidentified as cotton locusts (adult) with a corresponding error recognition rate of 4.40%.

## 4. Discussion

### 4.1. Comparison Results Summary

Automated entomology has been around for decades. Machine learning-based image processing models have been developed for accurate classification and identification of crop pests as traditional manual identification of insects is typically time-consuming, labor-intensive, and inefficient [19]. The classification accuracy mainly depends on input features. For early models such as support vector machine (SVM), neural nets, decision trees, k-nearest neighbor, all extracted features (e.g., shape, geometry, color, texture) often need to be redesigned and handcrafted, hence, image segmentation and feature extraction process needs to be integrated to transform the raw data into a suitable internal representation or feature vector, which increases the computational complexity of models [23,24,25,26,27,28,42,43]. Further, in many studies [30,31,32,44,45], the classification accuracy improved by applying deep learning, which performs automatic feature extraction from raw data that reduces the challenges of handcrafting features and can be effective in solving more complex problems. In Chen et al. [29] research, the classification of 16 kinds of common pests was performed by applying a deep learning model. The accuracy of most deep learning models is higher than 80%. In Wen et al. [45], an improved pyramidal stacked de-noising auto-encoder architecture is proposed to build a deep neural network for moth identification with identification accuracy of 96.9%. Xie et al. [32] proposes a multiple-kernel learning method for the classification of 24 common pest species of field crops and produces a high accuracy of approximately 97.20%. Thenmozhi et al. [30] propose an efficient deep CNN model to classify insect species on three publicly available insect datasets [31,32] which achieves an accuracy of 96.75%, 97.47%, and 95.97% on the three datasets, respectively. However, most of these studies take common pests (e.g., phalaenae, beetles, galleriidae) except locust of specific crops or vegetables (e.g., rice, maize, rape, soybean) as research objects. There are relatively few studies that involve the identification of locusts with different instars. According to our results, the identification accuracy of adult AM locust, rice locust, and cotton locust can reach 93.80%, 93.60%, and 97.80%, respectively, which are comparable with the above research results [29,30,31,32,45]. It is worthy of note that our sample images were directly collected in the field with diverse backgrounds, without employing lab-based settings (e.g., pheromone traps).

The identification accuracy of third-instar AM locusts is relatively low (77.20%). This is mainly because larvae and adults of AM locust are similar in morphological characteristics. Because recognizing AM locusts at an early stage is important in preventing their spread, the ResNet-Locust-BN shows a significant advantage in the identification of third-instar AM locust compared with AlexNet, GoogLeNet, ResNet 18, ResNet 50 and VggNet. Lu and Ye [42] propose the use of Grab Cut method, principal component analysis to design and extract eight features from 73 optional features of locust and then input the extracted features to SVM models (SVM type: C-support vector classification) to classify *Locusta migratoria manilensis* (AM locust) and *Oedaleus decorus asiaticus* with different instars. From the results they obtained, identification accuracy of fourth-instar and fifth-instar AM locust reached 95.63% and 95.78%, respectively. Nevertheless, complicated image segmentation and feature extraction need to be executed for each input sample, which makes it difficult to achieve fully automatic identification of locusts and instars using this method. Therefore, the ResNet-Locust-BN is more practical for classifying locusts with different species and instars than previously proposed methods.

### 4.2. Shortage and Future Work

This study is a preliminary attempt to explore the whether batch normalization based ResNet50 model can be applied in automatic classification of AM locusts, rice locusts, and cotton locusts, and to identify the growth status information of the AM locust (third-instar, fifth-instar, and adult) with high accuracy. In our future work, we aim to overcome several challenges for realizing the engineering application of locusts’ instars, species, and distribution information acquisition in an automatic manner. First and foremost, YOLOv2 network based model will be developed and integrated into the locust species and instars identification workflow so that the manual labeling can be replaced by automated target detection in the sample blocks generation phase. Secondly, integrated application of multispectral, hyperspectral, and laser point cloud observation technology will be explored to ascertain their ability to enhance the accuracy of locust sample detection and identification in complex backgrounds, e.g., plants, soil, and rocks, different types of insects, and other important elements. Thirdly, this study only addresses the automatic identification of AM locusts (third-instar, fifth-instar, and adult), rice locusts (adult), and cotton locusts (adult) as examples. In possible follow-up studies, it would be necessary to enrich the locust species and instars sample datasets to improve the automatic collection ability of the model. Fourthly, the five CNN models used in the comparison experiment are a bit old, thus, we will explore the possibility of using newer models such as InceptionNet [46], DensNet [47], ResNeXt [48], and SE-Net [10] to perform comparative experiments. Further, our future work will focus on developing a device that can be deployed in the field to automatically collect and transmit information concerning the species, instars, and distribution density of locusts. On this basis, and driven by a large number of quadrat-scale observation data [49], the relation between locust distribution density and the community structure changes in different regions habitats (water and heat conditions and vegetative growth state) can be evaluated as well as how to realize an integrated application of locust quadrant observation data and multisource satellite remote-sensing data.

## 5. Conclusions

In this paper, the ResNet-Locust-BN model was proposed and applied to locust species and instars identification problems. Based on ResNet architecture, we introduced BN function before each convolution layer to improve the model’s stability, robustness, convergence speed, and classification accuracy. Subsequently, locust image data collected in the field were manually labeled, extracted, and used as input samples to train the classifier parameters. Further, through comparison experiments, ReLU was chosen as the best activation function for the model and the initial learning rate, as well as batch size, were set to 0.1 and 32, respectively. The accuracy comparative experiments performed show that the proposed ResNet-Locust-BN model can effectively distinguish AM locusts, rice locusts, and cotton locusts and identify the growth status information of the AM locust (third-instar, fifth-instar, and adult) with high accuracy. The overall accuracy is up to 90.16%, which is higher than CNN models such as AlexNet, GoogLeNet, ResNet 18, ResNet 50, and VggNet. Also, the model shows good robustness and fast convergence rate.

## Figures and Tables

**Table 1:** Sample data sets for classification experiments.

No.	Species	Instars	Training Set Size	Validation Set Size	Testing Set Size
1	AM Locust	3	4000	1000	1000
2	AM Locust	4	4000	1000	1000
3	AM Locust	adult	4000	1000	1000
4	Rice Locust	adult	4000	1000	1000
5	Cotton Locust	adult	4000	1000	1000

**Table 2:** Analysis of the phenotypic characteristics of East Asian migratory (AM) locusts, rice locusts, and cotton locusts.

Species	Instar	Antennae Segments	Length (mm)	Coloring
AM locust	3	20–21	10–20	Gregarious: black; Dispersed: gray-green, brown-green;
AM locust	5	24–25	26–40	Gregarious: head reddish-brown, back black-brown, back foot diameter node light yellow Dispersed: in the color of the environment;
AM locust	adult	-	35–52	Gregarious: reddish-brown; Dispersed: mostly green, following the environmental color, overall body color is lighter;
Rice locust	adult	-	-	Green-brown green, or brownish back, side green;
Cotton locust	adult	-	45–81	Yellowish green, hind wings are rose;

**Table 3:** Comparison of the classification accuracy of several mainstream CNN models of locusts.

No.	CNN Model	Accuracy	No.	CNN Model	Accuracy
1	AlexNet	73.68%	4	ResNet50	80.84%
2	GoogLeNet	69.12%	5	VggNet	80.70%
3	ResNet18	67.60%	-	-	-

**Table 4:** Environment of the experimental infrastructure.

Hardware Facilities		Software Environment	
Server configuration	Intel^®^ Xeon(R) CPU E3-1230 v2^@^ 3.50 GHz, 32 GB memory	Operating system	Ubuntu 16.04
Graphics processing unit model	11 GB GTX 1080Ti	Plug-ins	Caffe, CUDA 8.0, cuDNN 5.1
Image acquisition device	Canon E.O.S. 5D Mark II	Development environment	JetBrains PyCharm, Python
